# Prevalence of Small A-Delta Fiber Neuropathy in Sjögren’s Disease: Findings from a Cohort Study

**DOI:** 10.3390/ijms262412013

**Published:** 2025-12-13

**Authors:** Magdalena Chylińska, Iga Kościńska-Shukla, Liwia Grudzień, Marta Jaskólska, Adam Wyszomirski, Natalia Dułak, Magdalena Rytlewska, Bartosz Karaszewski

**Affiliations:** 1 Department of Adult Neurology, Medical University of Gdańsk, 80-214 Gdańsk, Poland; adam.wyszomirski@gumed.edu.pl (A.W.); bartosz.karaszewski@gumed.edu.pl (B.K.); 2 Department of Internal Medicine, Connective Tissue Diseases and Geriatrics, Medical University of Gdańsk, 80-214 Gdańsk, Poland; iga.shukla@gmail.com (I.K.-S.); jaskolskamm@gmail.com (M.J.); ndulak@uck.gda.pl (N.D.); mrytlewska@uck.gda.pl (M.R.); 3 Department of Adult Neurology, University Clinical Center, 80-214 Gdańsk, Poland; liwiastrozek@gmail.com

**Keywords:** primary Sjögren’s disease, cutaneous silent period, small fiber neuropathy, neurophysiology

## Abstract

Polyneuropathy is a common condition that limits the quality of life among patients with primary Sjögren disease (pSjD). Somatic sensory fiber neuropathy involving small myelinated (A-δ) and unmyelinated C fibers may precede the development of sicca syndrome. The cutaneous silent period (CSP) is an inhibitory spinal reflex that can be used as a tool for evaluating the dysfunction of A-δ fibers. This study sought to examine CSP parameters, and their correlates, in patients with pSjD vs. healthy controls. We recruited 134 consecutive patients with a diagnosis of pSjD, of whom 109 subjects were included in the analysis. Electrodiagnostic tests comprised a nerve conduction study (NCS) and CSP analysis, alongside laboratory tests and questionnaires (the ESSPRI and SF-36). The examination of the healthy control (HC) group consisted of 113 NCSs and CSP studies. NCS tests of the median nerve in both groups were within the normal range. Statistical analysis revealed a significant difference in CSP duration (*p* < 0.001), S1 latency (*p* < 0.001) and S2 latency (*p* < 0.001) between the pSjD and HC groups. We observed prolonged CSP duration in approximately 38% of patients with pSjD and prolonged S2 latency in 18.35%. Small A-delta fiber neuropathy was diagnosed in 38% (41 subjects) patients. A regression analysis of CSP parameters indicated an association between the age of patients and PM Scl-75 antibodies (ab) levels in the pSjD cohort. As a new, noninvasive method of assessing A-δ nerve fibers, CSP was found to have a relation to the age and PM Scl-75 antibodies in patients with pSjD. The utility and sensitivity of CSP as a test for screening A-δ fiber function require further investigation in large cohorts of the pSjD population.

## 1. Introduction

Primary Sjögren’s disease (pSjD) is a chronic autoimmune disorder characterized by lymphocytic infiltration in the exocrine glands, primarily affecting the salivary and lacrimal glands [1]. Approximately 80% of patients exhibit a triad of symptoms, dryness of the mouth and eyes, fatigue, and joint pain, which severely affect quality of life (QOL) [2]. This condition may occur either in isolation or alongside organ-specific autoimmune diseases, such as thyroiditis or primary biliary cholangitis, in which case it is referred to as pSjD [2]. Immune complexes resulting from persistent B-cell hyperreactivity may induce extraepithelial manifestations, including peripheral neuropathy [2,3]. The most common polyneuropathy types are sensimotor axonal polyneuropathy and pure sensory polyneuropathy, specifically small fiber neuropathy (SFN) and sensory ataxic neuropathy [4,5]. pSjD -associated small fiber sensory neuropathy is often asymmetrical or non-length-dependent [4,6]. Small-caliber nerve fibers are classified into thinly myelinated A-δ and unmyelinated C fibers [7]. Aδ fibers transmit cold sensations, contribute to both cold and mechanical nociception, and mediate preganglionic sympathetic and parasympathetic cholinergic functions [8]. A previous study of Birnbaum et al. reported a higher prevalence of SFN in male patients with PSS [9]. The clinical picture of SFN includes neuropathic pain, diminished sensation to pinprick and temperature, and hyperesthesia, along with preserved deep tendon reflexes, vibration sense, and normal results from nerve conduction studies (NCSs) [4,10]. Its diagnosis involves several techniques that are relatively difficult to implement in routine clinical settings, such as quantitative sensory testing (QST), sympathetic skin response (SSR), the quantitative sudomotor axon reflex test (QSART) and skin biopsy [8]. The latter, utilized with a measurement of intraepidermal nerve fiber density (IENFD), is a sensitive method that predominantly assesses epidermal fibers originating from the dorsal root ganglia, which are presumed to represent the terminals of C and possibly A delta nociceptors [8,11]. However, a skin biopsy from the calf region is an invasive procedure mainly used for the diagnosis of a length-dependent phenotype [12]. When associated with an immunological disease, SFN may manifest in a non-length-dependent pattern, with a proximal, patchy, asymmetrical, or diffuse sensory distribution [8]. Devigili et al. (2008) proposed diagnostic criteria for SFN based on the presence of at least two abnormal findings among the following: clinical signs of small-fiber impairment (pinprick and thermal sensory loss and/or allodynia and/or hyperalgesia); abnormal warm and/or cold detection thresholds at the foot as assessed by QST; and reduced intraepidermal nerve fiber density (IENFD) at the distal leg [13,14]. Exclusion criteria included any clinical sign of large-fiber dysfunction (such as impaired light touch, reduced vibratory sensation, abnormal deep tendon reflexes, or limb/gait ataxia) as well as any abnormality detected on nerve conduction studies (NCSs) [13,14]. In vivo confocal microscopy (IVCM) assessment of nerve plexus density represents another non-invasive method for evaluating SFN in pSjD [15,16,17]. The cutaneous silent period (CSP) is an advanced neurophysiological parameter that reflects the functional integrity of hypomyelinated Aδ sensory fibers modulated by the corticospinal tract [18]. It is an inhibitory spinal reflex mediated by an afferent pathway of small-diameter A-δ fibers and an efferent pathway mediated by α-motoneurons [19,20]. It is assumed that the early component of CSP is mediated by group III (Aδ) fibers, while the late component of CSP results from the activity of group II (Aβ) fibers, particularly associated with cutaneous mechanoreceptors in the fingertips [21]. Suppressing surface electromyographic activity is achieved through a high-intensity, long-duration stimulation of cutaneous branches of sensory nerves during voluntary muscle contraction [22]. CSP analysis has been found to be a reliable neurophysiological method for assessing the function of A-δ small nerve fibers [23]. and was introduced to evaluate thin-fiber neuropathy in diabetes mellitus [24,25], Fabry disease [26] and HIV-related polyneuropathy [27]. CSP latency reflects the conduction function of A-delta afferents, efferent motor axons and synaptic delays, and the duration of the CSP depends on the amount of activated fibers and changes in nociceptive input [23]. A shorter duration of the CSP and delayed S1 latency are consistent with the involvement of small fiber neuropathy [19].

The aim of our study was to estimate the damage to small A-delta nerve fibers using CSP parameters in a cohort of patients with pSjD. We also evaluated clinical and laboratory factors associated with S1, S2 and CSP values in the studied population.

## 2. Results

**Figure 1:**
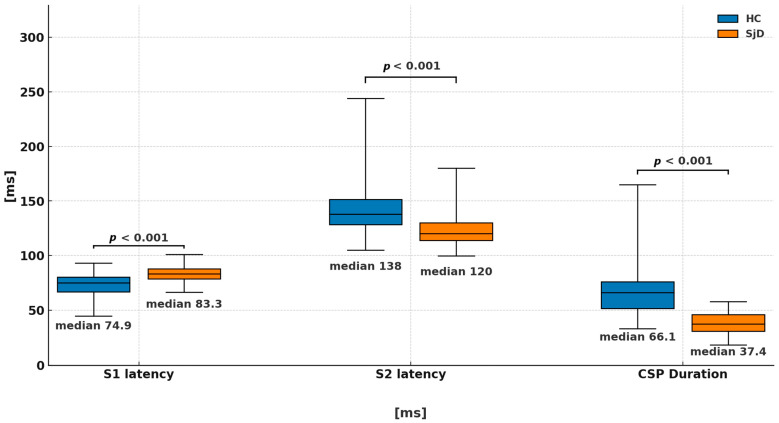
Significant differences in the parameters of S1 and S2 latency and CSP duration between the groups HC and pSjD group are demonstrated.

We studied 134 consecutive patients diagnosed with pSjD from the Department of Internal Diseases, Connective Tissue Diseases and Geriatrics of the Medical University of Gdansk, Poland. In total, 25 patients were excluded, meaning that 109 patients were included in the statistical analysis. The main demographic, clinical and electrophysiological characteristics of the studied populations are summarized in Table 1. The laboratory parameters of the study population are presented in Table 2. Detailed characteristics of the pSjD patient population presented in Table S1. The mean duration of the disease was 6 years, and the median age of patients was 55 years. The most common therapies applied to patients were steroids, hydroxychloroquine, azathioprine and methotrexate (Table 1). Moderate intensity of neuropathic pain was observed (mean 4.5/10) of the pSjD population, ranging from 3 to 7 on a 0–10 scale. Conduction parameters in the median nerve were within the normal range in both the pSjD and Hc groups. No statistically significant differences were observed in amplitude or conduction velocity in motor and sensory fibers of the median nerve; however, a significant difference was noted in median nerve motor latency (MNML) and F-wave latency to the detriment of the HC group. We observed a statistically significant difference between the pSjD and HC groups (Table 1 and Figure 1) in S1 latency (*p* < 0.001), S2 latency (*p* < 0.001) and CSP duration (*p* < 0.001). Reference limits were established according to the results obtained in the HC group, with the upper and lower normal limits of S1 and S2 latency and CSP duration (Table 1). S1 latency values exceeded the ULN in 18.4% of patients with pSjD, and the duration of the CSP was greater than the ULN in 38% of patients. Based on the presence of neuropathic symptoms and the absence of abnormalities in routine NCS and physical examination, and presence of abnormalities in CSP, we diagnosed A-delta fiber neuropathy in 38% of the pSjD patients (41 subjects) [12,28,29]. Table 3 provides a comprehensive summary of the clinical and electrophysiological parameters used for the diagnosis of A-delta fiber neuropathy in patients with pSjD.

**Figure 2:**
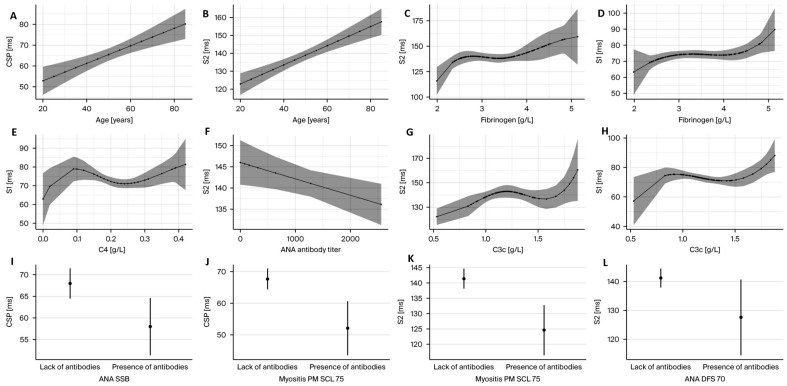
A graphical overview of the relationships between electrophysiological parameters and clinical or laboratory variables in the pSjD population. (**A**,**B**) indicate that age is a strong linear determinant of CSP duration and S2 latency. (**C**,**D**) depict that S2 and S1 latencies increase with higher fibrinogen concentrations. (**E**,**G**,**H**) show the associations between S1 and S2 latency and complement components C3c and C4 levels. (**F**) demonstrate an negative relationship between ANA titers and S2 latency. (**I**,**J**) indicate that the presence of anti-SSB/La ab and PMSCL75 ab is associated with reduced CSP duration. (**K**,**L**) demonstrate that presence of PMSCL75 ab and DFS 70 ab is associated with reduced S2 latency and CSP duration.

Based on the implemented regression models, an association between electrophysiological parameters and clinical or laboratory metrics was found (Figure 2). In the Supplementary Materials section, Table S2 presents all univariate linear regression models for the outcome variables (S1, S2, and CSP). The GEE model analysis revealed a significant association between age and S2 latency (*p* < 0.001, adjusted *p* < 0.001) and CSP duration (*p* < 0.001, adjusted *p* = 0.026) and between S2 latency and PM Scl-75 ab (*p* = 0.002, adjusted *p* = 0.067) after the Benjamini–Hochberg FDR correction. We observed a trend between S1 latency and complement C3c concentrations (*p* = 0.005, adjusted *p* = 0.011) and CSP duration and PM Scl-75 ab (*p* = 0.006, adjusted *p* = 0.11). Our analysis indicates that S2 and S1 latencies tend to increase with higher fibrinogen concentrations; however, this finding was not statistically significant. Furthermore, our results suggest that the presence of anti-Ro52, anti-SSB/La, PM/Scl-75, and DFS70 antibodies is associated with reduced CSP duration, although this association did not reach statistical significance.

## 3. Discussion

Neuropathy is a common finding in pSjD and can even precede sicca syndrome [30]. According to the literature, several forms of neuropathy in pSjD have been distinguished: sensory ataxic neuropathy, painful sensory neuropathy without sensory ataxia, multiple mononeuropathy, multiple cranial neuropathy, trigeminal neuropathy, autonomic neuropathy and radiculoneuropathy [31].

In the current study, we aimed to evaluate the CSP as a marker of SFN, assessing a large population of patients with pSjD using laboratory (biochemical), immunological and neurophysiological tests. The results demonstrated that the pSjD cohort had longer S1 and S2 latency and CSP duration than the HC group. The former exhibited normal conduction parameters (latency and conduction velocity) of motor and sensory fibers and normal F-wave latency. Accordingly, we propose that the presence of increased S1 and S2 latency and prolonged CSP duration reflects the underlying dysfunction of small fibers. Comparable observations have been reported regarding human immunodeficiency virus-related peripheral neuropathy [27]. Referring to recent studies carried out by Serrao et al. (2001) and Corsi et al. (2002), the abnormalities observed in patients with pSjD may be associated with the dysfunction of high-threshold A-δ fibers [32,33].

Previous researchers have discerned the prevalence of SFN in patients with pSjD to be around 9.2% using various diagnostic methods, including QST, skin biopsy and laser-evoked potentials [34]. Our results markedly exceed this percentage; we observed that 38% of the studied population presented abnormal CSP values and small A-delta fiber neuropathy. This finding is more consistent with a recent report conducted by Mori et al. [31].

Moreover, S2 latency and CSP duration are strongly related to age, consistent with Kaddah et al.’s study on healthy individuals [35]. Gemignani et al. proposed the notion that increasing age is a risk factor for the development of polyneuropathy in patients with pSjD, possibly induced by microangiopathic changes in the endoneurial vessels [36]. Sène et al.’s study on the prevalence of SFN in primary Sjögren’s syndrome (PSS) is particularly noteworthy: patients with SFN were older and had a higher incidence of arthralgia and xerostomia than those without SFN [37,38]. Moreover, patients with SFN were characterized by a lower frequency of serum markers of B-cell activation, antinuclear, anti-SSA, and anti-SSB antibodies, rheumatoid factor, and hypergammaglobulinemia [37]. This finding supports the results of our analysis that there is no obvious relationship between the aforementioned inflammatory parameters and the presence of SFN without ataxia in patients with pSjD. It is interesting to note that, in our study, no relationship was observed between the duration of the disease and the CSP parameters, which aligns with the research findings of Gono et al. [39] Regarding how autoantibodies contribute to the development of SFN, we observed that the presence of PM Scl-75 ab was associated with S2 latency and CSP duration. PM Scl-75 ab is classified as a myositis-associated autoantibody and is often found in SSc overlap syndromes [40]. Autoantigens associated with polymyositis–scleroderma overlap syndrome (PM/Scl) are nucleolar multiprotein particles, which possibly participate in the maturation of 5.8S rRNAs [41] and play a role in ribosome synthesis [42]. The PM/Scl antigen consists of at least 11 polypeptides, with relative molecular masses ranging from 20,000 to 110,000 [43]. The autoimmune reaction initiated by cellular damage or apoptosis leads to the release of exosome components and serves as a mechanism of PM/Scl autoreactivity. Given the central role of the human exosome in the processing and degradation of multiple RNA species [43], it is important to probe the extent to which the disruption of its function may affect normal cellular physiology and be implicated in the development of neuropathies. We identified such an association in this study and it should be validated in further studies on a larger cohort of patients with pSjD. Font et al. suggested that sensory polyneuropathy in pSjD could be related to antinuclear antibodies (ANAs) and anti-Ro/Sjogren’s syndrome A (SSA); however, we did not observe such a correlation in the current study [44].

According to the literature, there may be several mechanisms that underlie damage to the peripheral nervous system in patients with PSS. In some studies, vascular or perivascular inflammatory infiltrates, with or without necrosis, have been observed in peripheral nerve biopsy samples [45], and neurons may experience secondary effects from an inflammatory process that involves the *vasa nervorum* [45]. The immunopathogenesis of peripheral neuropathy (PN) has been well documented in other clinical conditions, such as in patients with anti-Hu or anti-sulfatide antibodies. Therefore, several antibodies reactive to proximal regions of sensory and motor neurons may contribute to PN in patients with PSS [45]. In sural nerve biopsies, a marked loss of fibers in sensory ataxic neuropathy and mild loss of fibers in painful sensory neuropathy are typically found. Vasculitis is most commonly observed in cases of mononeuritis multiplex. Autopsy findings in patients with sensory ataxic and painful neuropathy demonstrate neuronal loss in the dorsal root and sympathetic ganglia, accompanied by CD8-positive cytotoxic T lymphocytes [46]. Moreover, it has been postulated by Sène et al. and Jamilloux et al. that cryoglobulins may contribute to the development of sensory neuropathy in PSS [37,47]. In our cohort, we did not observe such a finding.

To summarize our findings, we discerned that SFN is relatively common in patients with pSjD. However, due to the design limitations of our study, the results should be interpreted with caution. The utility and sensitivity of the CSP as a diagnostic test of SFN dysfunction in the pSjD population should be further studied on larger groups of patients.

## 4. Materials and Methods

### 4.1. Patients

In this prospective study, we enrolled 134 patients diagnosed with PSS according to the 2016 ACR-EULAR Classification Criteria for Primary Sjögren’s Syndrome [48]. This study is a continuation of a larger project evaluating neurological complications among patients with pSjD [49,50]. Those with secondary SS, large fiber neuropathy, and diabetes were excluded (25 subjects). All patients underwent a clinical evaluation by a rheumatologist, and data on their medical history were collected. Overall, 109 patients with pSjD were ultimately included in the final analysis. The control group consisted of 113 age- and sex-matched healthy adults, who were members of the hospital staff. The procedures performed in this study were in accordance with the Declaration of Helsinki and approved by the Institutional Review Board (IRB) of the Medical University of Gdańsk, Poland (13 September 2024, no KB/438/2024). Written informed consent was obtained from all the participants involved in this study.

### 4.2. Neurophysiological Assessment

**Figure 3:**
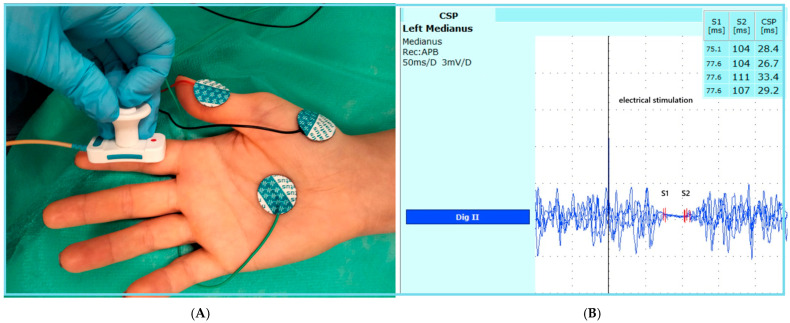
(**A**). The methodology of the CSP study; The electrode with the green cable is the ground electrode, the electrode with the black cable is the recording electrode, and the electrode with the red cable is the reference electrode. (**B**). A representative CSP from the healthy control group, S1 presents the onset latency, S2 presents the end latency, CSP represents the difference between the S2 and S1 latency values.

All examinations were conducted in the Adult Neurology Clinic, under standardized EMG laboratory conditions, with participants positioned horizontally. All CSP and NCS assessments were conducted by a single experienced investigator (L.G.) under rigorously standardized testing conditions. Limb temperature was maintained above 35 °C and verified using a certified Deymed Diagnostic thermometer, DEYMED Diagnostic s.r.o. Kudrnacova 533, 54931 Hronov, Czech Republic. All recordings exhibiting artifacts were excluded from the final analysis. No sedative medications were administered to any of the patients prior to or during the examinations. The EMG laboratory was acoustically and electrically shielded to minimize external interference. The electrophysiological tests were performed using a Dantec Keypoint G4 EMG/NCS/EP Workstation. We performed a routine motor and sensory median nerve conduction study and F-wave assessment on all subjects according to the American Association of Neuromuscular & Electrodiagnostic Medicine (AANEM) guidelines [51]. The CSP was elicited by a single electrical impulse (0.5 ms duration and 75–90 mA intensity, 250 ms sweeps, 30 and 10 kH filters) at the palmar side tip of the second digit using a bipolar stimulating electrode. Superficial tab electrodes (Natus Disposable Pre-Gelled Surface Electrodes, Natus Neurology Incorporated3150 Pleasant View Road, Middleton, WI, USA) were placed over the abductor pollicis brevis (ABP) muscle belly (Figure 3A), and electrical stimulation was performed during the near-maximum activation of the APB muscle. Near-maximum APB EMG activity was monitored and recorded using surface electrodes throughout the examination. A minimum of four consecutive responses were recorded and superimposed for analysis. The onset latency (S1) was defined as the point marking the beginning of muscle activity suppression, whereas the end latency (S2) corresponded to the onset of renewed muscle activity. The interval between these two latencies represented the duration of the cutaneous silent period (CSP) (Figure 3B).

### 4.3. Laboratory Test

Blood laboratory tests were performed in a certified laboratory of the University Clinical Center in Gdańsk using serum obtained from venous blood samples. Detailed methodologies for each laboratory assay are provided in the following links: https://uck.pl/content/download/2024/04/Z_11_CLK_KATALOG_CALOSC_ost.pdf (accessed on 1 December 2025). Routine laboratory tests were performed, and immunological parameters were recorded in all patients, comprising C3 and C4 complements detected using nephelometry; ANA tested via IF using the HEp-2 cell substrate; and anti-Ro/SS-A, anti-SSB/La, anti-HMGCR, anti-cN1A, anti-Ro52, anti-PL 12, PL 7, anti-SRP, anti-Jo1, anti-PM Scl75, and anti-PM Scl100 antibodies, which were detected using Western blot. They additionally included the erythrocyte sedimentation rate (ESR); peripheral blood counts; and levels of c-reactive protein (CRP), β-2-microglobulin, vitamin B12, fibrinogen, Fe, ferritin, folic acid, IgG, IgA, ASPAT, ALAT, GGTP, TSH, fT3, fT4, anti-TPO, anti-TG, vitamin B12, anti-CCP, and anti H.pylori.

### 4.4. Questionnaires

Participants were instructed to complete the Polish version of the EULAR Sjögren’s Syndrome Patient Reported Index (ESSPRI), and the Short Form Health Survey (SF-36) was employed to evaluate health-related quality of life (HR-QOL). The ESSPRI is a self-administered questionnaire used to assess the severity of symptoms such as dryness, fatigue, and pain on a 0–10 numerical scale; a score of 0 indicates the lowest intensity of the symptoms [52]. For the purposes of the current study, the pain domain was categorized into the three following types: neuropathic, muscular, and articular. Neuropathic pain was characterized as numbness, pins-and-needles sensations or a burning sensation in the upper or lower limbs. Muscular pain comprised aching or stiffness of the muscles or a feeling that the muscles have been overworked, and articular pain was determined as pain with/or without stiffness limited to joints. Pain was assessed similarly to the other symptoms, taking into account the preceding two weeks and using a numerical scale of 0–10. Based on the presence of neuropathic symptoms (numbness, pins-and-needles sensations or a burning sensation in the upper or lower limbs) and the absence of abnormalities in routine ENG and physical examination, and abnormal result CSP, we diagnosed A-delta fiber neuropathy (Table 3). The 36-Item Short Form Health Survey (SF-36) is the most widely applied instrument for assessing health-related quality of life (HR-QOL) in patients with rheumatic diseases. In the present study, the Optum SF-36v2 Health Survey was employed. To assess pSjD activity, the EULAR Sjögren’s Syndrome Disease Activity Index (ESSDAI) was scored by a rheumatologist and consisted of 12 domains assessing organ involvement and laboratory abnormalities [52].

### 4.5. Statistics

All statistical analyses were performed using R software (version 4.4.2). A two-sided *p*-value of <0.10 indicated statistical significance, reflecting the exploratory nature of this investigation. Continuous variables are summarized using the median with interquartile range (IQR) and minimum and maximum values, alongside the number of observations and missing values. Categorical variables are presented as frequencies, percentages, and the number of missing observations. H. pylori antibody values exceeding 200 units were truncated to 200 units (n = 5 observations). Univariate linear regression models were independently fitted for each outcome variable (S1, S2, and CSR), utilizing clinical variables, quantitative laboratory parameters, and semi-quantitative laboratory measures as predictors. Myositis-specific antibodies and antinuclear antibody (ANA) variables were analyzed as binary variables, indicating either the absence or presence of antibodies. To evaluate the assumption of linearity, a visual comparison was conducted between linear regression lines and locally weighted scatterplot smoothing (LOESS) curves. In instances where substantial deviations from linearity were observed, B-spline regression with 4 degrees of freedom was implemented. Residual normality was evaluated using the Shapiro–Wilk test. When normality assumptions were violated, generalized estimating equation (GEE) models with a Gaussian distribution, identity link function, and independence correlation structure were applied. The assumption of constant variance was tested using the studentized Breusch–Pagan test. In cases where heteroscedasticity was detected, models were fitted using generalized least squares (GLS) estimation. The independence assumption was considered satisfied based on the study design. An analysis of variance (ANOVA) was performed to ascertain whether predictors significantly explained the variability in outcomes. The results were reported as F-statistics with numerator degrees of freedom, denominator degrees of freedom, and corresponding *p*-values. To mitigate the issue of multiple comparisons, *p*-values were adjusted using the Benjamini–Hochberg false discovery rate (FDR) correction method. A complete case analysis approach was utilized, wherein missing values were excluded from all statistical analyses. Selected models were visualized by displaying regression predictions with 90% confidence intervals to illustrate the relationship between predictors and outcomes. Differences between the pSjD and HC groups were evaluated using the permuted Yuen–Welch *t*-test. This analysis employed 1000 replications and applied 10% trimming to address issues of unequal variances and outliers.

## 5. Conclusions

To the best of our knowledge, this is the first study of a large cohort of patients with pSjD that offers a detailed examination of CSP parameters in relation to wide range of biochemical and immunological tests. Although numerous laboratory parameters were analyzed, the pathogenesis of SFN and diagnostic methods require further investigation.

Limitation of the Study: We did not correct CSP duration and S1 and S2 latency by length of extremity or height of the patients, and we did not assess QST and symptoms of dysautonomia in the studied population. In the examined pSjD patient population IENF assessments were not performed, which we acknowledge as a limitation of the present study. Future research on small-fiber neuropathy in pSjD should include adjustments for patient height, which may influence electrophysiological and neurophysiological parameters, and should also incorporate objective measures of thermal sensory thresholds and validated dysautonomia tests to improve diagnostic accuracy and phenotypic characterization.

## Figures and Tables

**Table 1:** Demographic, clinical and neurophysiological characteristics of the study’s participants.

Parameter	pSjD Patients	Healthy Controls (HCs)	*p*-Value ^1^
Age [y]			
Median (Q1–Q3); min–max	55.0 (43.0–65.0); 23.0–82	54.0 (45.0–64.0); 24.0–76	0.861
	*n* = 109	*n* = 113	
Sex			
F/M (n/%)	104/5 (95.4/4.6%)	107/6 (94.7/5.3%)	
Disease duration [y]			
Median (Q1–Q3); min–max	6.0 (3.0–9.0); 1.0–66, 1.0	n.a	
ESSPRI neuropathic symptoms			
Median (Q1–Q3); min–max	4.5 (3.0–7.0); 0.0–10	n.a	
	Diagnostic score		
Mean; min–max	4.5 (4.0, 6.0)	n.a	
	Dry eyes and/or dry mouth positive n/%		
	86/79%	n.a	
	Salivary gland biopsy positive n/%		
	88/81%	n.a	
	anti-Ro/SSA positive n/%		
	52/48%	n.a	
	anti-La/SSB positive n/%		
	21/20%	n.a	
Therapy history n/%			
Steroids	78/71.56%	n.a	
Hydroxychloroquine	62/56.88%	n.a	
Azathioprine	24/22%	n.a	
Methotrexate	21/19.27%	n.a	
Cyclophosphamide	9/8.26%	n.a	
Cyclosporine	3/2.75%	n.a	
Mycophenolate mofetil	2/1.84%	n.a	
IVIG	2/1.84%	n.a	
Rituximab	5/4.59%	n.a	
Neurophysiological parameters			
S1 latency [ms]			
Median (Q1–Q3); min–max	74.9 (66.8–80.2); 44.7–93	83.3 (78.7–87.8); 66.6–101	<0.001
LLN, ULN [ms]		65.05, 101.45	
S2 latency [ms]			
Median (Q1–Q3); min–max	138.0 (128.3–151.3); 105.0–244	120.0 (114.0–130.0); 99.6–180	<0.001
LLN, ULN [ms]		90, 154	
CSP [ms]			
Median (Q1–Q3); min–max	66.1 (51.5–76.0); 33.3–165	37.4 (31.0–46.0); 18.4–58	<0.001
LLN, ULN [ms]		25, 68.4	
Median nerve conduction parameters			
CMAP amp. [mV], mean (SD)	7.11 (1.79)	7.50 (1.75)	0.76
MNCV m/s, mean (SD)	55.96 (4.22)	55.8 (4.17)	0.069
MNDL ms, mean (SD)	3.02 (0.42)	3.39 (0.34)	<0.001
SNAP amp. [uV], mean (SD)	21.89 (11.26)	19.73 (8.92)	0.11
SNAP CV [m/s], mean (SD)	58.05 (6.59)	57.53 (6.12)	0.43
SNAP lat. [ms], mean (SD)	2.57 (0.29)	2.64 (0.34)	0.13
F-wave latency [ms]	23.22 (2.93)	23.97 (1.89)	0.04

Abbreviations: pSjD: primary Sjögren’s disease; n: number of patients; F: female; M: male; y: years; ESSPRI: EULAR Sjögren’s Syndrome Disease Activity Index; S1: onset latency; S2: end latency; n.a.: not applicable; Q: quartile; CSP: cutaneous silent period; LLN: lower limit of normal; ULN: upper limit of normal; IVIG: intravenous immunoglobulin; SD: standard deviation; ms: milliseconds; CMAP: compound motor action potential; amp: amplitude; MNCV: motor nerve conduction velocity; MNDL: motor nerve distal latency; SNAP: sensory nerve action potential; CV: conduction velocity; lat.: latency. A *p*-value of 0.05 is considered significant. ^1^ The permutation-based Yuen–Welch *t*-test.

**Table 2:** Laboratory parameters of pSjD patients.

	Parameter
	Folic acid ng/mL
Median (Q1–Q3); Min–Max	7.6 (6.0–12.8), 0.0–22
	Gammaglobulins g/L
Median (Q1–Q3); Min–Max	14.7 (13.1–17.1), 6.9–39,
	AspAT U/L
Median (Q1–Q3); Min–Max	25.0 (21.0–30.0), 13.0–49
	AlAT U/L
Median (Q1–Q3); Min–Max	21.0 (16.0–27.5), 6.0–64
	GGTP U/L
Median (Q1–Q3); Min–Max	18.0 (15.0–24.0), 8.0–238
	CRP mg/L
Median (Q1–Q3); Min–Max	0.9 (0.5–1.7), 0.0–21
	ESR mm/h
Median (Q1–Q3); Min–Max	11.0 (5.0–19.0), 2.0–108
	Creatinine mg/dL
Median (Q1–Q3); Min–Max	0.8 (0.7–0.9), 0.4–2
	GFR mL/min/m^2^
Median (Q1–Q3); Min–Max	90.0 (80.5–90.0), 31.0–90
	Fibrinogen g/L
Median (Q1–Q3); Min–Max	3.4 (2.8–3.7), 2.0–5
	Ferritin ng/mL
Median (Q1–Q3); Min–Max	56.5 (33.1–92.5), 2.4–409
	Vitamin B12 pg/mL
Median (Q1–Q3); Min–Max	412.0 (333.0–549.0), 38.0–1, 175
	IgA g/L
Median (Q1–Q3); Min–Max	2.2 (1.7–3.0), 0.5–5
	anti-CCP U/mL
Median (Q1–Q3); Min–Max	0.0 (0.0–0.6), 0.0–268
	Beta-2 Microglobulin mg/L
Median (Q1–Q3); Min–Max	1.8 (1.5–2.2), 1.1–6
	C3c g/L
Median (Q1–Q3); Min–Max	1.2 (1.1–1.3), 0.5–2
	C4 g/L
Median (Q1–Q3); Min–Max	0.2 (0.2–0.3), 0.0–0.4
	H.pylori antibodies U/mL
Median (Q1–Q3); Min–Max	13.2 (6.2–39.1), 0.0–200
	TSH uU/mL
Median (Q1–Q3); Min–Max	1.1 (0.8–1.5), 0.0–6
	fT3 pmol/L
Median (Q1–Q3); Min–Max	4.3 (4.0–4.6), 0.7–21
	fT4 pmol/L
Median (Q1–Q3); Min–Max	12.5 (11.5–13.3), 9.5–31
	anti-TPO IU/mL
Median (Q1–Q3); Min–Max	0.0 (0.0–10.5), 0.0–972
	anti-TG IU/mL
Median (Q1–Q3); Min–Max	0.0 (0.0–11.0), 0.0–4, 809
	Fe µ/dL
Median (Q1–Q3); Min–Max	92.0 (70.0–115.0), 25.0–176

Abbreviations: pSjD: primary Sjögren’s disease; AspAT: Aspartate Aminotransferase; AlAT: Alanine Aminotransferase; GGTP: Gamma-Glutamyl Transferase; CRP: C-Reactive Protein; ESR: Erythrocyte Sedimentation Rate; GFR: Glomerular Filtration Rate; IgA: Immunoglobulin A; anti-CCP: Anti-Cyclic Citrullinated Peptide Antibodies; C3c: Complement component 3c; C4: Complement component 4; TSH: Thyroid-Stimulating Hormone; fT3: Free Triiodothyronine; fT4: Free Thyroxine; anti-TPO: anti-Thyroid Peroxidase Antibodies; anti-TG: anti-Thyroglobulin Antibodies; Fe: Iron.

**Table 3:** Comprehensive summary of the clinical and electrophysiological parameters used for the diagnosis of A-delta fiber neuropathy in patients with pSjD.

Parameter	pSjD Patients, n (%)
Clinical symptoms (ESSPRI neuropathic): numbness, pins-and-needles sensations, a burning sensation in the upper or lower limbs	75 (68.8%)
Electrophysiology parameters Latency S1 (ms) CSP duration (ms)	values outside the reference limits 41 (37.6%) 41 (37.6%)
NCS (median nerve) CMAP amp. [mV] MNCV [m/s] MNDL [ms] SNAP lat. [ms] SNAP amp. [µV] SNAP CV [m/s]	above LLN >5 mV (100%) >50 m/s (100%) below ULN <4.2 (100%) <3.4 (100%) above LLN >10 (100%) >50 (100%)
Physical examination motor/sensory deterioration KJ AJ	abnormal findings 0 (0%) 0 (0%) 0 (0%)

Abbreviations: pSjD: primary Sjögren’s disease; ESSPRI: EULAR Sjögren’s Syndrome Disease Activity Index; CSP: cutaneous silent period; S1: onset latency NCS: nerve conduction study; CMAP: compound motor action potential; amp: amplitude; MNCV: motor nerve conduction velocity; MNDL: motor nerve distal latency; SNAP: sensory nerve action potential; CV: conduction velocity; lat.: latency; LLN, Lower Limit of Normal; ULN, Upper Limit of Normal; KJ; knee jerk; AJ; ankle jerk.

## Data Availability

The raw data supporting the conclusions of this article will be made available by the authors on request.

## Supplementary Materials

The following supporting information can be downloaded at: https://www.mdpi.com/article/10.3390/ijms262412013/s1.
