# Supplementary material for: Prevalence of Small A-Delta Fiber Neuropathy in Sjögren’s Disease: Findings from a Cohort Study
Source: Int J Mol Sci. 2025 Dec 13;26(24):12013. doi: 10.3390/ijms262412013 (PMC12732339; doi:10.3390/ijms262412013)
Supplement: Supplementary file 1 [file ijms-26-12013-s001.zip › Table S1.pdf]

Table S1. Clinical and laboratory data of PSjD group.

Variable	N=109
S1	
Median (Q1-Q3); Min-Max; N missing	75.0 (66.8-80.6),44.7-93, 0.0
S2	
Median (Q1-Q3); Min-Max; N missing	138.0 (129.0-151.3),105.0-244, 0.0
CSP	
Median (Q1-Q3); Min-Max; N missing	66.1 (53.0-76.0),33.3-165, 0.0
Disease duration	
Median (Q1-Q3); Min-Max; N missing	6.0 (3.0-9.0),1.0-66, 1.0
Age	
Median (Q1-Q3); Min-Max; N missing	55.0 (43.0-65.0),23.0-82, 0.0
Gender	F 104/5
	95,4/4,6%
ESSPRI neuropathic	
Median (Q1-Q3); Min-Max; N missing	4.5 (3.0-7.0),0.0-10, 73.0
folic acid	
Median (Q1-Q3); Min-Max; N missing	7.6 (6.0-12.8),0.0-22, 0.0
Gammaglobulins	
Median (Q1-Q3); Min-Max; N missing	14.7 (13.1-17.1),6.9-39, 7.0
AspAT	
Median (Q1-Q3); Min-Max; N missing	25.0 (21.0-30.0),13.0-49, 2.0
AlAT	
Median (Q1-Q3); Min-Max; N missing	21.0 (16.0-27.5),6.0-64, 1.0
GGTP	
Median (Q1-Q3); Min-Max; N missing	18.0 (15.0-24.0),8.0-238, 3.0
CRP	
Median (Q1-Q3); Min-Max; N missing	0.9 (0.5-1.7),0.0-21, 1.0
ESR	
Median (Q1-Q3); Min-Max; N missing	11.0 (5.0-19.0),2.0-108, 6.0
Creatinine	
Median (Q1-Q3); Min-Max; N missing	0.8 (0.7-0.9),0.4-2, 1.0
GFR	
Median (Q1-Q3); Min-Max; N missing	90.0 (80.5-90.0),31.0-90, 1.0
Fibrinogen	
Median (Q1-Q3); Min-Max; N missing	3.4 (2.8-3.7),2.0-5, 3.0
Fe	
Median (Q1-Q3); Min-Max; N missing	92.0 (70.0-115.0),25.0-176, 1.0
Ferritin	
Median (Q1-Q3); Min-Max; N missing	56.5 (33.1-92.5),2.4-409, 0.0
vitamin B12	
Median (Q1-Q3); Min-Max; N missing	412.0 (333.0-549.0),38.0-1,175, 0.0
IgA	
Median (Q1-Q3); Min-Max; N missing	2.2 (1.7-3.0),0.5-5, 7.0
anti-CCP	
Median (Q1-Q3); Min-Max; N missing	0.0 (0.0-0.6),0.0-268, 3.0
B2MG	
Median (Q1-Q3); Min-Max; N missing	1.8 (1.5-2.2),1.1-6, 1.0
C3c	

Median (Q1-Q3); Min-Max; N missing	1.2 (1.1-1.3),0.5-2, 8.0
C4	
Median (Q1-Q3); Min-Max; N missing	0.2 (0.2-0.3),0.0-0.4, 8.0
H.pylori antibodies	
Median (Q1-Q3); Min-Max; N missing	13.2 (6.2-39.1),0.0-200, 5.0
ANA, n / N (%); N missing	
0	2 / 104 (1.9%); 5
80	2 / 104 (1.9%); 5
160	11 / 104 (10.6%); 5
320	22 / 104 (21.2%); 5
640	22 / 104 (21.2%); 5
1280	10 / 104 (9.6%); 5
2560	35 / 104 (33.7%); 5
TSH	
Median (Q1-Q3); Min-Max; N missing	1.1 (0.8-1.5),0.0-6, 2.0
fT3	
Median (Q1-Q3); Min-Max; N missing	4.3 (4.0-4.6),0.7-21, 5.0
fT4	
Median (Q1-Q3); Min-Max; N missing	12.5 (11.5-13.3),9.5-31, 4.0
anti-TPO	
Median (Q1-Q3); Min-Max; N missing	0.0 (0.0-10.5),0.0-972, 3.0
anti-TG	
Median (Q1-Q3); Min-Max; N missing	0.0 (0.0-11.0),0.0-4,809, 3.0
myositis HMGCR, n / N (%); N missing	
0	102 / 107 (95.3%); 2
1	3 / 107 (2.8%); 2
2	1 / 107 (0.9%); 2
3	1 / 107 (0.9%); 2
myositis cN1A, n / N (%); N missing	
0	98 / 108 (90.7%); 1
1	3 / 108 (2.8%); 1
2	4 / 108 (3.7%); 1
3	3 / 108 (2.8%); 1
myositis Ro52, n / N (%); N missing	
0	72 / 107 (67.3%); 2
1	2 / 107 (1.9%); 2
2	7 / 107 (6.5%); 2
3	26 / 107 (24.3%); 2
myositis OJ, n / N (%); N missing	
0	105 / 107 (98.1%); 2
1	2 / 107 (1.9%); 2
myositis EJ, n / N (%); N missing	
0	107 / 107 (100.0%); 2
myositis PL012, n / N (%); N missing	
0	107 / 107 (100.0%); 2
myositis PL07, n / N (%); N missing	
0	105 / 107 (98.1%); 2
1	2 / 107 (1.9%); 2
myositis SRP, n / N (%); N missing	
0	105 / 107 (98.1%); 2

1	2 / 107 (1.9%); 2
myositis Jo01, n / N (%); N missing	
0	107 / 107 (100.0%); 2
myositis PM0Scl75, n / N (%); N missing	
0	101 / 107 (94.4%); 2
1	5 / 107 (4.7%); 2
2	1 / 107 (0.9%); 2
myositis PM0Scl100, n / N (%); N missing	
0	98 / 107 (91.6%); 2
1	8 / 107 (7.5%); 2
3	1 / 107 (0.9%); 2
myositis Ku, n / N (%); N missing	
0	103 / 106 (97.2%); 3
1	2 / 106 (1.9%); 3
2	1 / 106 (0.9%); 3
myositis p/SAE1, n / N (%); N missing	
0	106 / 107 (99.1%); 2
1	1 / 107 (0.9%); 2
myositis NXP2, n / N (%); N missing	
0	107 / 107 (100.0%); 2
myositis MDA5, n / N (%); N missing	
0	106 / 107 (99.1%); 2
1	1 / 107 (0.9%); 2
myositis TIFg, n / N (%); N missing	
0	106 / 107 (99.1%); 2
3	1 / 107 (0.9%); 2
myositis Mi02 beta, n / N (%); N missing	
0	100 / 107 (93.5%); 2
1	6 / 107 (5.6%); 2
2	1 / 107 (0.9%); 2
myositis Mi02 alfa, n / N (%); N missing	
0	106 / 107 (99.1%); 2
3	1 / 107 (0.9%); 2
ANA DFS70, n / N (%); N missing	
0	100 / 106 (94.3%); 3
3	6 / 106 (5.7%); 3
ANA PCNA, n / N (%); N missing	
0	106 / 106 (100.0%); 3
ANA gp210, n / N (%); N missing	
0	104 / 106 (98.1%); 3
1	1 / 106 (0.9%); 3
2	1 / 106 (0.9%); 3
ANA RP155, n / N (%); N missing	
0	105 / 106 (99.1%); 3
1	1 / 106 (0.9%); 3
ANA RP11, n / N (%); N missing	
0	104 / 106 (98.1%); 3
1	2 / 106 (1.9%); 3
ANA PM/Scl75, n / N (%); N missing	
0	100 / 106 (94.3%); 3

1	5 / 106 (4.7%); 3
2	1 / 106 (0.9%); 3
ANA PM/Scl100, n / N (%); N missing	
0	99 / 106 (93.4%); 3
1	5 / 106 (4.7%); 3
2	1 / 106 (0.9%); 3
3	1 / 106 (0.9%); 3
ANA Scl70, n / N (%); N missing	
0	105 / 106 (99.1%); 3
1	1 / 106 (0.9%); 3
ANA PML, n / N (%); N missing	
0	106 / 106 (100.0%); 3
ANA Sp100, n / N (%); N missing	
0	105 / 106 (99.1%); 3
1	1 / 106 (0.9%); 3
ANA centrom B, n / N (%); N missing	
0	104 / 106 (98.1%); 3
2	1 / 106 (0.9%); 3
3	1 / 106 (0.9%); 3
ANA centrom A, n / N (%); N missing	
0	103 / 106 (97.2%); 3
1	2 / 106 (1.9%); 3
3	1 / 106 (0.9%); 3
ANA Ku, n / N (%); N missing	
0	104 / 106 (98.1%); 3
1	1 / 106 (0.9%); 3
2	1 / 106 (0.9%); 3
ANA Mi02 beta, n / N (%); N missing	
0	100 / 107 (93.5%); 2
1	5 / 107 (4.7%); 2
2	1 / 107 (0.9%); 2
3	1 / 107 (0.9%); 2
ANA Mi02 alfa, n / N (%); N missing	
0	104 / 106 (98.1%); 3
1	1 / 106 (0.9%); 3
2	1 / 106 (0.9%); 3
ANA Sm, n / N (%); N missing	
0	105 / 106 (99.1%); 3
1	1 / 106 (0.9%); 3
ANA nRNP/Sm, n / N (%); N missing	
0	103 / 106 (97.2%); 3
1	2 / 106 (1.9%); 3
3	1 / 106 (0.9%); 3
ANA SSB, n / N (%); N missing	
0	94 / 106 (88.7%); 3
1	3 / 106 (2.8%); 3
2	4 / 106 (3.8%); 3
3	5 / 106 (4.7%); 3
ANA Ro52, n / N (%); N missing	
0	71 / 107 (66.4%); 2

1	4 / 107 (3.7%); 2
2	6 / 107 (5.6%); 2
3	26 / 107 (24.3%); 2
ANA SSA, n / N (%); N missing	
0	74 / 107 (69.2%); 2
1	3 / 107 (2.8%); 2
2	3 / 107 (2.8%); 2
