# Supplementary material for: Prevalence of Small A-Delta Fiber Neuropathy in Sjögren’s Disease: Findings from a Cohort Study
Source: Int J Mol Sci. 2025 Dec 13;26(24):12013. doi: 10.3390/ijms262412013 (PMC12732339; doi:10.3390/ijms262412013)
Supplement: Supplementary file 1 [file ijms-26-12013-s001.zip › Table S2.pdf]

Table S2. Univariate linear regression models for outcome variable (S1, S2, and CSR), utilizing clinical variables, quantitative laboratory parameters, and semi-quantitative laboratory measures as predictors.

Model	Outcome	Predictor	Regression type	F statistic (df1, df2)	P-value	Adjusted p-value
Model 1	S1	disease duration	Linear regression with B-spline	0.18 (1, 103)	0.674	0.857
Model 2	S2	disease duration	GEE model with B-spline	1.62 (1, 103)	0.206	0.636
Model 3	CSP	disease duration	GEE model with B-spline	1.19 (1, 103)	0.279	0.717
Model 4	S1	Age	Linear regression	2.69 (1, 107)	0.104	0.560
Model 5	S2	Age	GEE model	22.55 (1, 107)	<0.001	<0.001
Model 6	CSP	Age	GEE model	12.85 (1, 107)	<0.001	0.026
Model 7	S1	ESSPRI neuropathic	Linear regression with B-spline	0.82 (1, 31)	0.371	0.759
Model 8	S2	ESSPRI neuropathic	GEE model with B-spline	2.27 (1, 31)	0.142	0.636
Model 9	CSP	ESSPRI neuropathic	GEE model with B-spline	1.59 (1, 31)	0.217	0.636
Model 10	S1	folic acid	Linear regression with B-spline	1.50 (1, 104)	0.224	0.636
Model 11	S2	folic acid	GEE model with B-spline	0.35 (1, 104)	0.554	0.834
Model 12	CSP	folic acid	GEE model with B-spline	0.00 (1, 104)	0.953	0.982
Model 13	S1	Gammaglobulins	Linear regression with B-spline	0.03 (1, 97)	0.858	0.921
Model 14	S2	Gammaglobulins	GEE model with B-spline	0.05 (1, 97)	0.817	0.915
Model 15	CSP	Gammaglobulins	GEE model with B-spline	0.09 (1, 97)	0.764	0.901
Model 16	S1	AspAT	Linear regression with B-spline	0.51 (1, 102)	0.477	0.786
Model 17	S2	AspAT	GEE model with B-spline	2.56 (1, 102)	0.113	0.560
Model 18	CSP	AspAT	GEE model with B-spline	1.57 (1, 102)	0.213	0.636
Model 19	S1	GGTP	Linear regression with B-spline	0.71 (1, 101)	0.403	0.783
Model 20	S2	GGTP	GEE model with B-spline	0.52 (1, 101)	0.471	0.786
Model 21	CSP	GGTP	GEE model with B-spline	0.07 (1, 101)	0.786	0.901
Model 22	S1	CRP	Linear regression with B-spline	0.10 (1, 103)	0.748	0.896

Model 23	S2	CRP	GEE model with B-spline	0.66 (1, 103)	0.418	0.786
Model 24	CSP	CRP	GEE model with B-spline	0.84 (1, 103)	0.362	0.759
Model 25	S1	ESR	Linear regression with B-spline	0.08 (1, 98)	0.783	0.901
Model 26	S2	ESR	GEE model with B-spline	2.87 (1, 98)	0.093	0.560
Model 27	CSP	ESR	GEE model with B-spline	1.95 (1, 98)	0.166	0.636
Model 28	S1	GFR	Linear regression with B-spline	0.60 (1, 104)	0.442	0.786
Model 29	S1	Fibrinogen	Linear regression with B-spline	2.82 (1, 101)	0.096	0.560
Model 30	S2	Fibrinogen	GEE model with B-spline	4.02 (1, 101)	0.048	0.504
Model 31	CSP	Fibrinogen	GEE model with B-spline	1.58 (1, 101)	0.211	0.636
Model 32	S1	Fe	Linear regression with B-spline	0.28 (1, 103)	0.601	0.834
Model 33	S2	Fe	GEE model with B-spline	0.07 (1, 103)	0.796	0.901
Model 34	CSP	Fe	GEE model with B-spline	0.00 (1, 103)	0.991	0.991
Model 35	S1	Ferritin	Linear regression with B-spline	0.37 (1, 104)	0.545	0.834
Model 36	S2	Ferritin	GEE model with B-spline	0.97 (1, 104)	0.326	0.759
Model 37	CSP	Ferritin	GEE model with B-spline	0.37 (1, 104)	0.545	0.834
Model 38	S1	vitamin B12	Linear regression with B-spline	0.23 (1, 104)	0.634	0.834
Model 39	S2	vitamin B12	GEE model with B-spline	2.65 (1, 104)	0.107	0.560
Model 40	CSP	vitamin B12	GEE model with B-spline	1.87 (1, 104)	0.174	0.636
Model 41	S1	IgA	Linear regression with B-spline	0.52 (1, 97)	0.471	0.786
Model 42	S2	IgA	GEE model with B-spline	0.00 (1, 97)	0.979	0.988
Model 43	CSP	IgA	GEE model with B-spline	0.24 (1, 97)	0.623	0.834
Model 44	S1	B2MG	Linear regression with B-spline	1.68 (1, 103)	0.197	0.636
Model 45	S2	B2MG	GEE model with B-spline	1.81 (1, 103)	0.181	0.636

Model 46	CSP	B2MG	GEE model with B-spline	0.00 (1, 103)	0.954	0.982
Model 47	S1	C3c	Linear regression with B-spline	8.18 (1, 96)	0.005	0.114
Model 48	S2	C3c	GEE model with B-spline	3.75 (1, 96)	0.056	0.522
Model 49	CSP	C3c	GEE model with B-spline	0.00 (1, 96)	0.963	0.982
Model 50	S1	C4	Linear regression with B-spline	4.42 (1, 96)	0.038	0.504
Model 51	S2	C4	GEE model with B-spline	0.02 (1, 96)	0.888	0.935
Model 52	CSP	C4	GEE model with B-spline	2.74 (1, 96)	0.101	0.560
Model 53	S1	H.pylori antibodies	GEE model with B-spline	0.04 (1, 99)	0.852	0.921
Model 54	S2	H.pylori antibodies	GEE model with B-spline	0.79 (1, 99)	0.375	0.759
Model 55	CSP	H.pylori antibodies	GEE model with B-spline	0.51 (1, 99)	0.477	0.786
Model 56	S1	ANA	Linear regression with B-spline	3.98 (1, 99)	0.049	0.504
Model 57	S2	ANA	GEE model	4.24 (1, 102)	0.042	0.504
Model 58	CSP	ANA	GEE model with B-spline	0.97 (1, 99)	0.328	0.759
Model 59	S1	TSH	Linear regression with B-spline	0.57 (1, 102)	0.451	0.786
Model 60	S2	TSH	GEE model with B-spline	2.24 (1, 102)	0.138	0.636
Model 61	CSP	TSH	GEE model with B-spline	1.00 (1, 102)	0.320	0.759
Model 62	S1	fT4	Linear regression with B-spline	0.80 (1, 100)	0.374	0.759
Model 63	S2	fT4	GEE model with B-spline	0.88 (1, 100)	0.350	0.759
Model 64	CSP	fT4	GEE model with B-spline	0.20 (1, 100)	0.657	0.846
Model 65	S1	myositis HMGCR (binary)	Linear regression	0.02 (1, 105)	0.890	0.935
Model 66	S2	myositis HMGCR (binary)	GEE model	0.31 (1, 105)	0.580	0.834
Model 67	CSP	myositis HMGCR (binary)	GEE model	0.12 (1, 105)	0.727	0.896
Model 68	S1	myositis cN1A (binary)	Linear regression	1.50 (1, 106)	0.223	0.636

Model 69	S2	myositis cN1A (binary)	GEE model	1.43 (1, 106)	0.235	0.636
Model 70	CSP	myositis cN1A (binary)	GEE model	0.24 (1, 106)	0.627	0.834
Model 71	S1	myositis Ro52 (binary)	Linear regression	0.12 (1, 105)	0.726	0.896
Model 72	S2	myositis Ro52 (binary)	GEE model	0.50 (1, 105)	0.483	0.786
Model 73	CSP	myositis Ro52 (binary)	GEE model	0.28 (1, 105)	0.596	0.834
Model 74	S1	myositis PM Scl75 (binary)	Linear regression	0.07 (1, 105)	0.792	0.901
Model 75	S2	myositis PM Scl75 (binary)	GEE model	10.11 (1, 105)	0.002	0.067
Model 76	CSP	myositis PM Scl75 (binary)	GEE model	8.02 (1, 105)	0.006	0.114
Model 77	S1	myositis PM Scl100 (binary)	Linear regression	2.62 (1, 105)	0.108	0.560
Model 78	S2	myositis PM Scl100 (binary)	GEE model	1.79 (1, 105)	0.184	0.636
Model 79	CSP	myositis PM Scl100 (binary)	GEE model	0.22 (1, 105)	0.639	0.834
Model 80	S1	myositis Mi 2 beta (binary)	Linear regression	0.11 (1, 105)	0.739	0.896
Model 81	S2	myositis Mi 2 beta (binary)	GEE model	0.91 (1, 105)	0.343	0.759
Model 82	CSP	myositis Mi 2 beta (binary)	GEE model	0.48 (1, 105)	0.489	0.786
Model 83	S1	ANA DFS70 (binary)	Linear regression	0.29 (1, 104)	0.593	0.834
Model 84	S2	ANA DFS70 (binary)	GEE model	2.81 (1, 104)	0.097	0.560
Model 85	CSP	ANA DFS70 (binary)	GEE model	3.49 (1, 104)	0.065	0.554
Model 86	S1	ANA PM/Scl75 (binary)	Linear regression	0.77 (1, 104)	0.383	0.759
Model 87	S2	ANA PM/Scl75 (binary)	GEE model	0.62 (1, 104)	0.432	0.786
Model 88	CSP	ANA PM/Scl75 (binary)	GEE model	1.58 (1, 104)	0.211	0.636
Model 89	S1	ANA PM/Scl100 (binary)	Linear regression	0.77 (1, 104)	0.382	0.759
Model 90	S2	ANA PM/Scl100 (binary)	GEE model	0.04 (1, 104)	0.839	0.921
Model 91	CSP	ANA PM/Scl100 (binary)	GEE model	0.24 (1, 104)	0.628	0.834

Model 92	S1	ANA Mi02 beta (binary)	Linear regression	0.11 (1, 105)	0.739	0.896
Model 93	S2	ANA Mi02 beta (binary)	GEE model	0.91 (1, 105)	0.343	0.759
Model 94	CSP	ANA Mi02 beta (binary)	GEE model	0.48 (1, 105)	0.489	0.786
Model 95	S1	ANA SSB (binary)	Linear regression	1.21 (1, 104)	0.274	0.717
Model 96	S2	ANA SSB (binary)	GEE model	1.69 (1, 104)	0.196	0.636
Model 97	CSP	ANA SSB (binary)	GEE model	4.91 (1, 104)	0.029	0.495
Model 98	S1	ANA Ro52 (binary)	Linear regression	0.31 (1, 105)	0.576	0.834
Model 99	S2	ANA Ro52 (binary)	GEE model	0.25 (1, 105)	0.620	0.834
Model 100	CSP	ANA Ro52 (binary)	GEE model	0.04 (1, 105)	0.841	0.921
Model 101	S1	ANA SSA (binary)	Linear regression	0.37 (1, 105)	0.544	0.834
Model 102	S2	ANA SSA (binary)	GEE model	2.54 (1, 105)	0.114	0.560
Model 103	CSP	ANA SSA (binary)	GEE model	1.46 (1, 105)	0.229	0.636

df1: the numerator degrees of freedom; df2: the denominator degrees of freedom; GEE: Generalized Estimating Equation.
